# Supplementary material for: Splice-Junction-Based Mapping of Alternative Isoforms in the Human Proteome
Source: Cell Rep. Author manuscript; Available in PMC 2020 Jan 15. (PMC6961840; doi:10.1016/j.celrep.2019.11.026)
Supplement: 2 [file NIHMS1546469-supplement-2.zip › DF1/PXD010154/Liver/Liver_35_MAPKAPK5_QVIEEQTTSHESTGGAK.pdf]

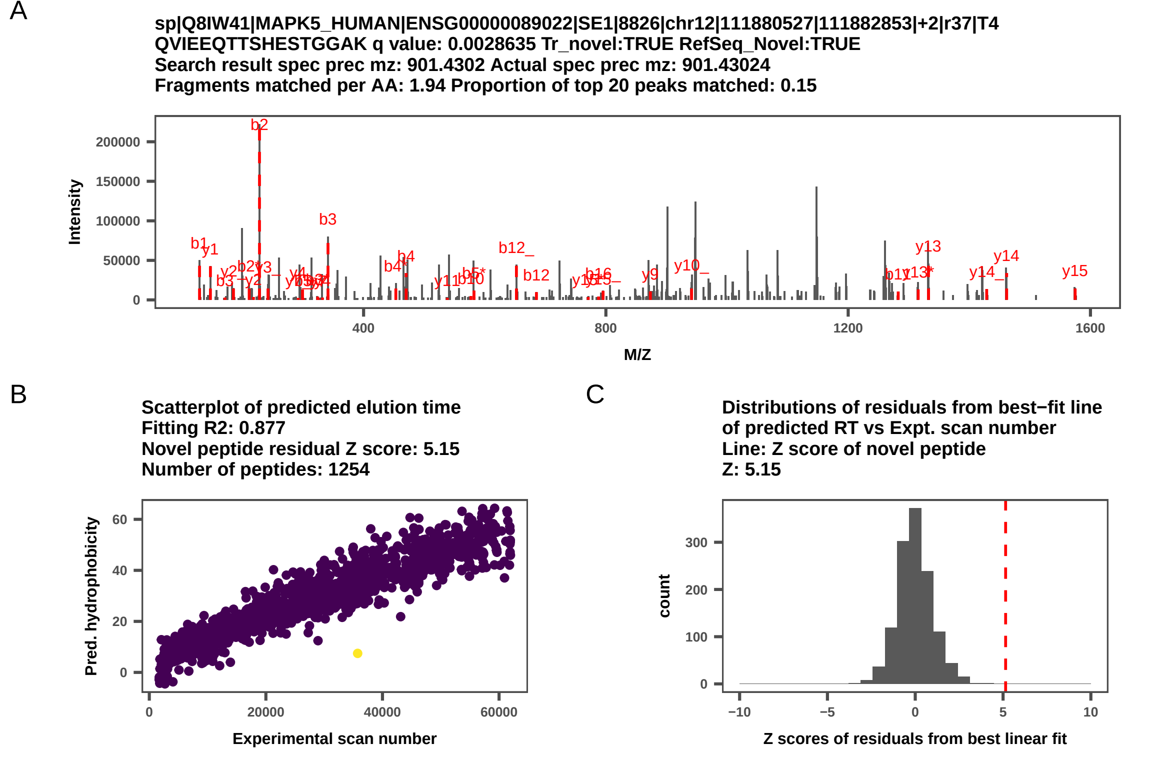
A
sp|Q8IW41|MAPK5_HUMAN|ENSG00000089022|SE1|8826|chr12|111880527|111882853|+2|r37|T4
QVIEEQTTSHESTGGAK q value: 0.0028635 Tr_novel:TRUE RefSeq_Novel:TRUE
Search result spec prec mz: 901.4302 Actual spec prec mz: 901.43024
Fragments matched per AA: 1.94 Proportion of top 20 peaks matched: 0.15
Intensity
200000
150000
100000
50000
0
b2
b3
b1
y1
b4
b12_
b12
y9
y10_
y13
y14
y14_
y15
400
800
1200
1600
M/Z
B
Scatterplot of predicted elution time
Fitting R2: 0.877
Novel peptide residual Z score: 5.15
Number of peptides: 1254
Pred. hydrophobicity
60
40
20
0
0
20000
40000
60000
Experimental scan number
C
Distributions of residuals from best−fit line
of predicted RT vs Expt. scan number
Line: Z score of novel peptide
Z: 5.15
count
300
200
100
0
−10
−5
0
5
10
Z scores of residuals from best linear fit
